# Probationers in Excess—II

**Published:** 1908-08-01

**Authors:** 


### Nursing Administration.

### PROBATIONERS IN EXCESS—II.

We have shown that in many hospitals the number of completely trained nurses falls considerably below 20 or even 25 per cent. A varying percentage of probationers, amounting in the case of the Nightingale probationers in 1907 to a third of the whole number, but usually not exceeding ten per cent., leave from one cause or another before completing their training, and this increases the proportion of new hands in the staff. Taking the matron, her assistants, and the sisters in some representative hospitals it is found that the number does not make up more than 12 or 15 per cent. of the whole number. This means that less than a quarter of the whole staff are prepared for full responsibility, and indicates that a severe strain is resting on both sisters and matron. That such a state of affairs may be produced at any time accidentally is due to the want of stability which still attends all women's occupations. The percentage of women who discontinue a career after having successfully begun it is still a large one. So far as we have been able to judge, ill-health bears a very small share in the losses of the nursing service. Family claims, inheritance, and marriage are the leading causes which induce people to break through a course of training which is in satisfactory progress. Every matron is exposed to disarrangement of her plans, and the proportion of new hands suddenly increased by unexpected resignations, and a change of matron often brings about so many changes in the upper ranks of the staff, that they can best be met by promoting all round, thus creating numerous vacancies for juniors. Still, when all these things are taken into consideration, it should be well understood by the governing body that such a condition of affairs ought to be purely exceptional. The percentage of untrained persons in the staff ought to be regularly checked, and should it show a tendency to rise in normal circumstances, to over 20 per cent. with a four years' course, or over 25 per cent. in a three years' course, steps should be taken to increase the number of sisters. The proportion of wholly untrained persons in the staff ought never to exceed in number the proportion of the completely trained and more permanent nurses, and we venture to maintain that where this balance is disturbed, a serious amount of worry, resulting from a sense of supervision unfulfilled, must be the portion of the matron.

In a previous article statistics were given showing the proportion of beds which fall on an average to each nurse in the night and day staff in certain general hospitals. These ought to be read in connection with the number of first-year probationers in the staff. In the big London training schools the percentage falls as low as 2.0 at St. Bartholomew's, 2.1 at the London and at Guy's, 2.3 at St. George's and at King's College Hospitals. In all these hospitals except at St. George's paying pupils are received, their number amounting to as many as 31 at Guy's. It is in the nature of things reasonable that in hospitals receiving a large number of pupils the percentage of beds to each member of the ward staff should be low. A junior probationer will spend two or three times as many hours over her work as an experienced nurse, besides absorbing half the attention of someone else to teach her. Where the number of first-year probationers is unusually high, and the number of beds to each member of the staff is not low in proportion, it may be assumed beyond question that a great strain is being put on the sisters, for it would be unfair to assume that the nursing of the hospital was allowed to suffer. In general, sisters trained in a good school will undergo almost any personal sacrifice to keep the nursing up to the mark in their particular ward, but they will not long stay in the service of such institutions as make good work impracticable without sacrifice of health and reasonable leisure. It will be seen, if the tables are examined, that where the proportion of first-year probationers in the staff is kept within limits, the number of beds to each member of the staff can be kept fairly high without undue strain to anyone concerned. Out of six provincial hospitals with medical schools quoted in the tables the proportion of junior probationers is under 25 per cent., sinking at the Liverpool Royal Southern to 17.6 per cent. In no one of them is the proportion of beds to nurses lower than 3.2; in two it is 3.5, and in one 3.8. The matter is one of more importance from the financial point of view than is generally understood. By some curious law which governs committees, expenditure on the staff is commonly regarded solely under the heading of salary. Governors would deem that they were recklessly wasting the money subscribed for charitable purposes if, by raising her salary fifty pounds, they retained the services of a matron who was saving them hundreds of pounds yearly by her excellent management; yet they will readily acquiesce in adding several new probationers to the staff, especially if such are to pay some small premium, although from first to last they must cost the institution a good sixty pounds a year each. Always striving to keep the salaries within bounds, the number of untrained workers is increased out of all proportion in some institutions, and the inevitable result is beheld in the gradually diminishing number of beds to each nurse. Every fraction is of importance in this calculation, as may be seen from the following example. Given a hospital with 150 beds in constant occupation, a staff of 50 nurses will be needed, of which five may be engaged principally outside the wards. The proportion of beds to each member of the ward staff will work out at 3.3—a very fair average. If the average were lowered to 2.5, ten extra nurses would be needed, at a cost of not less than £900 a year.

It is only possible to keep down the number of the staff, and at the same time to ensure the nursing being kept up to a high standard, by not employing too many nurses undergoing training. In the big training schools this is fully understood, and the staff is large in proportion to the teaching undertaken. In smaller institutions mistakes are, however, often made for want of a clear grasp of the situation.

